# Supplementary material for: Work-Function-Dependent Reduction of Transition Metal Nitrides in Hydrogen Environments
Source: J Phys Chem Lett. 2024 Nov 8;15(46):11462–7. doi: 10.1021/acs.jpclett.4c02259 (PMC11587081; doi:10.1021/acs.jpclett.4c02259)
Supplement: Supplementary file 1 — jz4c02259_si_001.pdf [file jz4c02259_si_001.pdf]

Work Function Dependent Reduction of Transition Metal Nitrides (TMNs) in Hydrogen Environments

Abdul Rehman[†*], Robbert W.E. van de Kruijs[†], Wesley T.E. van den Beld[†], Jacobus M. Sturm[†], and Marcelo Ackermann[†]

[†]*Industrial Focus Group XUV Optics, MESA+ Institute for Nanotechnology, University of Twente, Drienerlolaan 5, 7522NB Enschede, the Netherlands*

* E-mail: a.rehman@utwente.nl

1. ΔG for the reduction of TMN_x and TMO_y

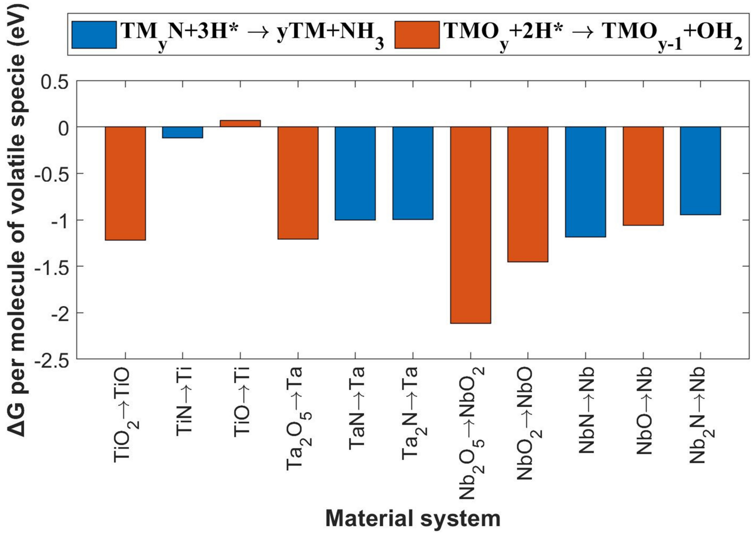

Figure S1. Change in the Gibbs free energy (ΔG) for the reduction reaction of TMN_x and TMO_y per molecule to volatile specie (NH_3 and H_2O, respectively) calculated at 700 °C and 0.02 mbar of working pressure.[1,2] We hypothesize that H_2O formation is energetically favorable on O-rich surface TMO_xN_y, since ΔG for the reduction reaction of TMO_y ($y>1$) is less than TMN_x ($x\leq1$). Whereas NH_3 formation is favorable over H_2O formation on N-rich surface TMO_xN_y.

2. Surface de-oxidation (O/TM)

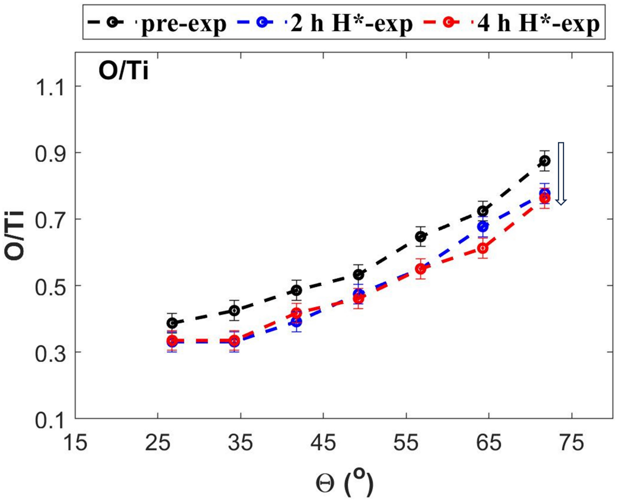

Figure S2. Change in the O/Ti ratio measured over the range of AR-XPS measurements in the pre- and post-H*-exposed TiN sample. The drop in the O/Ti ratio upon 2 h H*-exposure is due the de-oxidation of the surface TMO_xN_y.

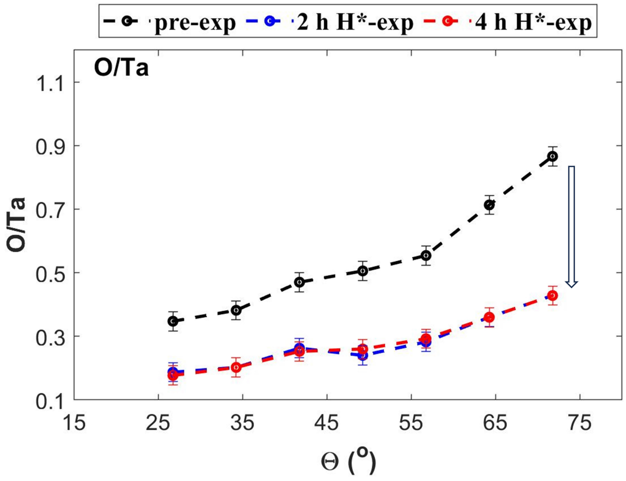

Figure S3. Change in the O/Ta ratio measured over the range of AR-XPS measurements in the pre- and post-H*-exposed TaN sample. The drop in the O/Ta ratio indicates de-oxidation of the surface TMO_xN_y.

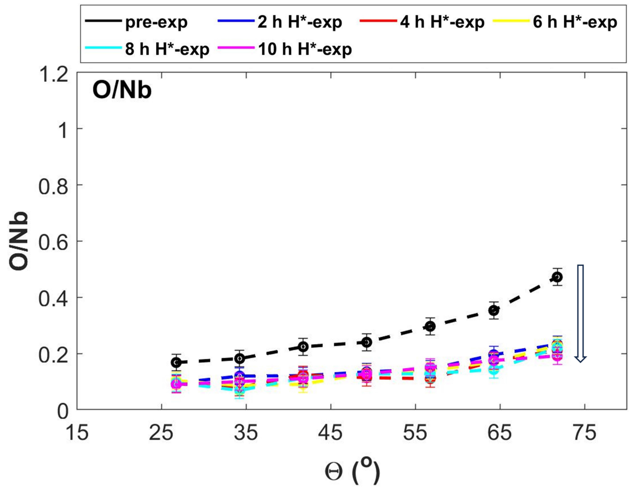

Figure S4. Change in the O/Nb ratio measured over the range of AR-XPS measurements in the pre- and post-H*-exposed NbN sample. De-oxidation of the surface TMO_xN_y resulted in a decrease in the O/Nb ratio.

3. Comparison of AR-XPS spectra

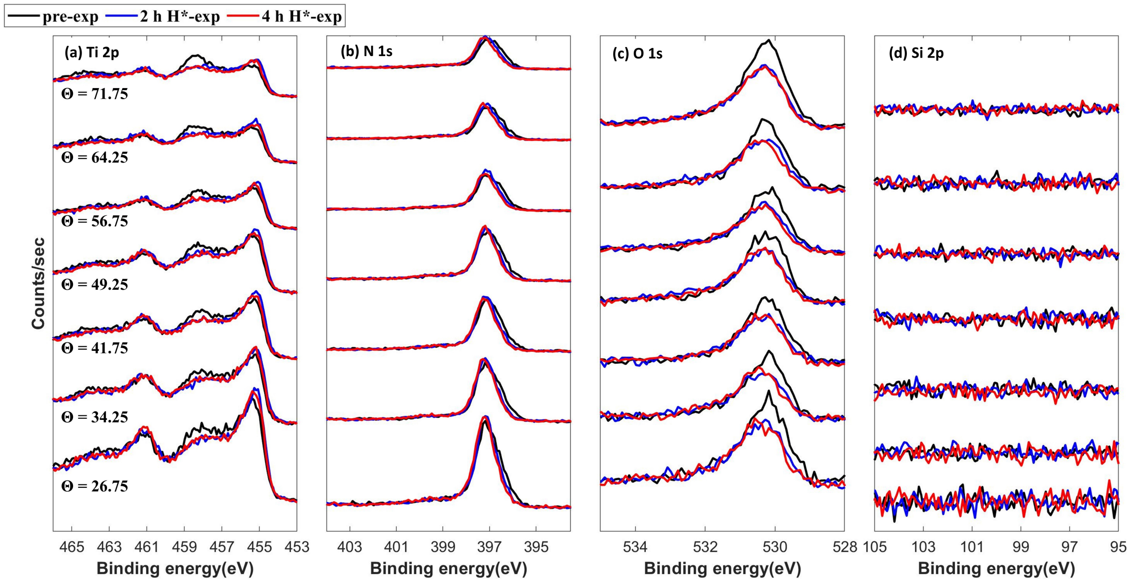

Figure S5. Comparison of the XPS spectra taken over the range of AR-XPS measurements of the pre-exposed (in black), 2 h H*-exposed (in blue), and 4 h H*-exposed (in red) TiN sample. (a) Ti 2p, (b) N 1s, (c) O 1s, and (d) Si 2p.

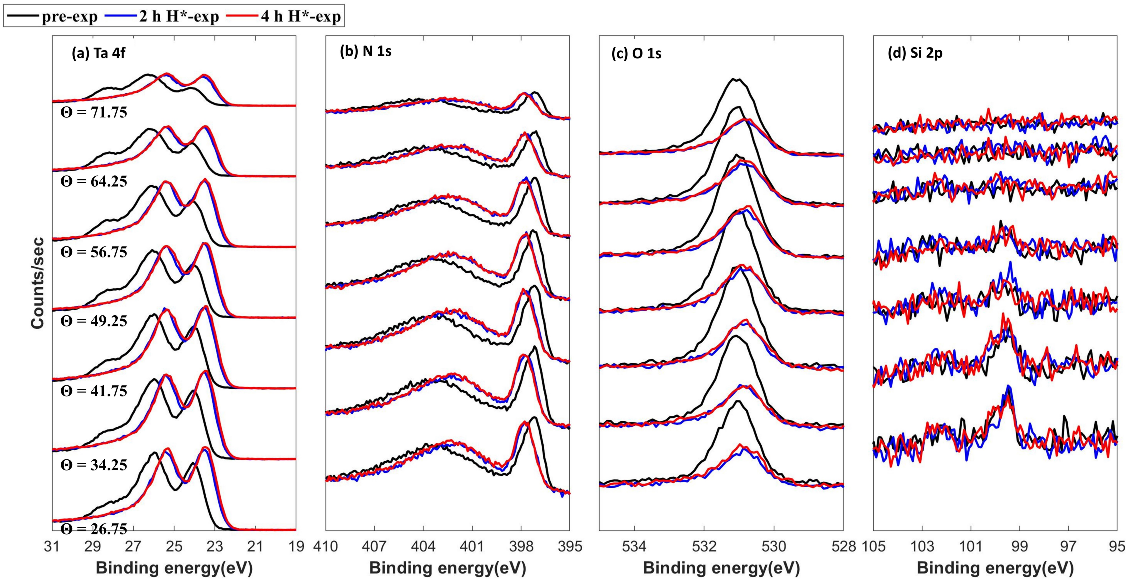

Figure S6. Comparison of the XPS spectra taken over the range of AR-XPS measurements of the pre-exposed (in black), 2 h H*-exposed (in blue), and 4 h H*-exposed (in red) TaN sample. (a) Ta 4f, (b) N 1s, (c) O 1s, and (d) Si 2p.

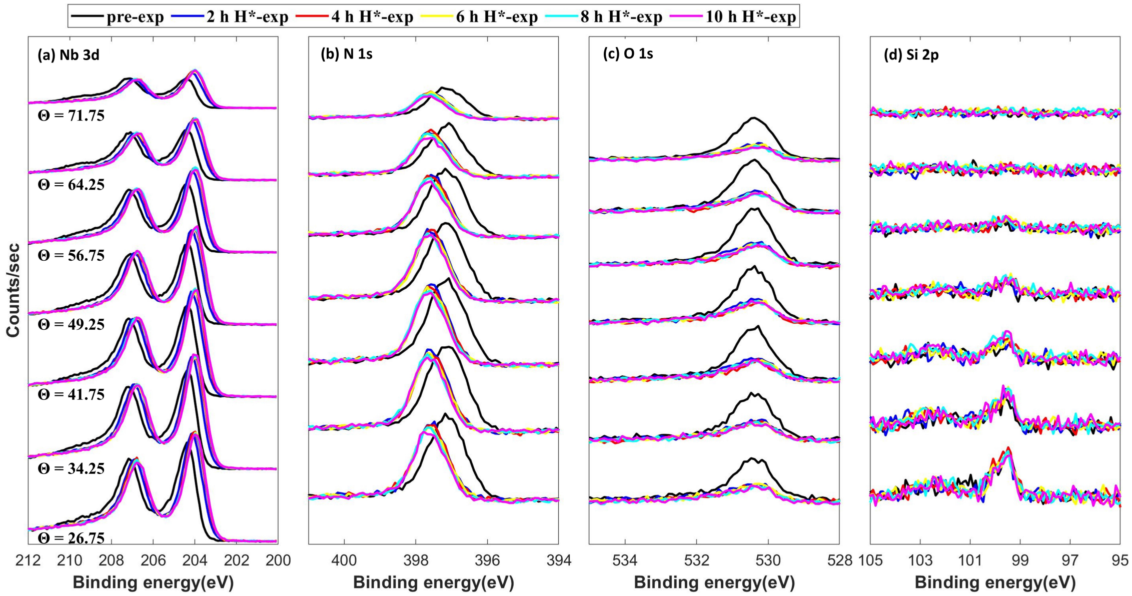

Figure S7. Comparison of the XPS spectra taken over the range of AR-XPS measurements of the pre-exposed (in black), 2 h H*-exposed (in blue), 4 h H*-exposed (in red), 6 h H*-exposed (in yellow), 8 h H*-exposed (in cyan), and 10 h H*-exposed (in magenta) NbN sample. (a) Nb 3d, (b) N 1s, (c) O 1s, and (d) Si 2p.

4. XPS spectra fitting

In this section, we outline the methodology used for fitting the core-level TM and N 1s XPS spectra.

For all the XPS spectra analyses, Shirley background subtraction was applied, and the spectra were fitted using Voigt profile doublets and peaks. We use Avantage software for spectral fitting.

In the following subsections, we detail the specific fitting constraints used for each sample, including the Gaussian-Lorentzian mix percentage (GL), peak positions, and peak areas.

4.1. TiN XPS spectra fitting

The Ti 2p XPS spectra were fitted with four doublets, each comprising Ti $2p_{3/2}$ and Ti $2p_{1/2}$ components. The separation between these components was constrained to 6.0 ± 0.2 eV,[3] and the area ratio (Ti $2p_{1/2}$/Ti $2p_{3/2}$) was fixed to 0.515.[4] GL for the TiN, TiN-sat, TiO_xN_y/TiO_x, and TiO_2 doublets was set to 80, 95, 30, and 60, based on the literature.[5]

For the conductive TiN doublet, which exhibits asymmetry, the tail exponent was left as a free parameter (resulting in a fitted value of 0.09), while the tail mix was fixed at 0 (note that tail exponent and mix are specific to the Avantage software). Additionally, the area of the Ti $2p_{3/2}$ component of the TiN-sat doublet was constrained to be 0.52 times the area of the TiN doublet's Ti $2p_{3/2}$ component.[4,5] The Ti $2p_{3/2}$ peak positions for the TiN and TiO_2 doublets was fixed at the binding energies of 455.2 ± 0.2 eV and 458.3 ± 0.2 eV, respectively.[5] For the TiN-sat doublet, the peak position of the Ti $2p_{3/2}$ component was constrained to be 3 eV above the Ti $2p_{3/2}$ peak position of the TiN doublet. The peak position of the Ti $2p_{3/2}$ component of the TiO_xN_y/TiO_x doublet was left as a free parameter, resulting is a fitted value of 456.5 eV binding energy.[5] Notably, no shift in the position of the doublets is observed from pre- to post-H* exposure.

N 1s XPS spectra of the TiN sample were fitted with two peaks with GL set to 90 for

the TiN/TiO_xN_y peak and 0 for the TiO_xN_y/TiN-sat peak.[5] In the pre-exposed sample, the TiN/TiO_xN_y and TiO_xN_y/TiN-sat peaks were fitted at 397.1 eV and 399.6 eV, respectively.[5] The TiN/TiO_xN_y peak is shifted by ≈ 0.2 eV higher binding energy in the 2 h H*-exposed sample. This is attributed to the surface de-oxidation. No further change in the N 1s spectra is observed upon addition 2 h (4 h H*-exp) H*-exposure.

The full width at half maximum (FWHM) of the fitted peaks and their respective areas are provided in the subsequent table (Table S1).

Table S1. Full Width at Half Maximum (FWHM) and area (CPS) in eV of the fitted peaks in the Ti 2p and N 1s spectra of the pre-exp, 2 h H*-exp, and 4 h H*-exp TiN sample, taken at $\Theta = 34.25°$.

Peak	Pre-exp		2 h H*-exp		4 h H*-exp	
	FWHM	Area CPS	FWHM	Area CPS	FWHM	Area CPS
Ti $2p_{3/2}$ TiN	0.9	323	0.9	342	0.9	348
Ti $2p_{3/2}$ TiN-sat	2.0	170	2.0	180	2.0	183
Ti $2p_{3/2}$ TiON/TiO	1.8	98	2.0	103	2.0	104
Ti $2p_{3/2}$ TiO_2	2.0	120	2.0	52	2.0	52
N 1s TiN/TiON	1.0	201	0.9	211	0.9	207
N 1s ad N/TiON	1.4	10	1.7	15	1.8	20

4.2. TaN XPS spectra fitting

The Ta 4f XPS spectra were fitted with three doublets (with Ta $4f_{7/2}$ and Ta $4f_{5/2}$ components) and one peak (O 2s). The separation between the components of the doublets was constrained to 2 ± 0.1 eV[6] and the area ratio (Ta $4f_{7/2}$/Ti $4f_{5/2}$) was fixed at 0.788.[4] The peak position of the Ta $4f_{7/2}$ components of the TaN and Ta_2O_5 doublets were constrained to 23.5 ± 1 eV and 26.5 ± 0.1 eV, respectively.[6,7] Furthermore, the peak position of O 2s was fixed at 23.6 ± 0.1 eV.[6] We also fix the amplitude of the O 2s peak to 5% of the measured O 1s peak intensity.[8] The peak position of the Ta $4f_{7/2}$ component in the TaO_xN_y/TaO_x doublet was left as a free parameter, resulting in a fit at 26.5 eV binding energy.[6]

Ta $4f_{7/2}$ component of the TaN doublet in the pre-H*-exposed sample is at 24.0 eV binding energy, while after 2 h H*-exposure, the doublet is shifted by ≈ 0.4 eV lower binding energy

due to the de-nitridation. No significant change in the Ta 4f XPS spectra is observed after 2 h H*-exposure.

The N 1s spectra of the TaN sample are fitted with two peaks, with a GL set to 30. The peak position of the Ta $4f_{7/2}$ was constrained to 403.0 ± 1 eV. The TaN/TaO_xN_y peak position in the pre-exposed sample is fitted at 397.2 eV binding energy.[6] Upon 2 H*-exposure the peak shifted by 0.4 eV higher binding energy due to the de-oxidation. Similar to the Ta 4f XPS spectra, no major change in the N 1s spectra of the sample is observed after 2 h H*-exposure.

Note that the O 2s contribution in the Ta 4f XPS spectra is negligible (less than 0.6% of the integrated Ta 4f intensity) and does not affect the calculated Ta fraction in the sample beyond the measurement uncertainty.

FWHM of the fitted peaks and their respective areas are provided in the subsequent table (Table S2).

Table S2. FWHM and area in eV of the fitted peaks in the Ta 4f and N 1s spectra of the pre-exp, 2 h H*-exp, and 4 h H*-exp TaN sample taken at $\Theta = 34.25°$.

Peak	Pre-exp		2 h H*-exp		4 h H*-exp	
	FWHM	Area CPS	FWHM	Area CPS	FWHM	Area CPS
Ta $4f_{7/2}$ TaN	1.0	882	1.2	1498	1.2	1574
Ta $4f_{7/2}$ TaO_xN_y	1.6	597	1.6	407	1.6	374
Ta $4f_{7/2}$ Ta_2O_5	1.7	584	1.7	166	1.6	153
O 2s	1.0	23	1.0	12	1.0	12
N 1s TaN_x	1.4	693	1.3	519	1.3	514
N 1s Ta $4p_{3/2}$	6.5	1603	6.9	1954	6.7	1902

4.3. NbN XPS spectra fitting

The Nb 3d XPS spectra were fitted with three doublets, each containing Nb $3d_{5/2}$ and Nb $3d_{3/2}$ components. The separation between these components was constrained to 2.8 ± 0.1 eV, with the fixed area ratio (Nb $3d_{3/2}$/Nb $3d_{5/2}$) of 0.689.[9] The peak positions of the Nb $3d_{5/2}$ components of the NbN and Nb_2O_5 doublets were constrained to 204.0 ± 0.3 eV and 207.2 ± 0.1 eV, respectively.[9] The Nb $3d_{5/2}$ peak position of the NbO_xN_y/NbO_x doublet

was treated as a free parameter, yielding a fit at 205.5 eV binding energy.[9]

Nb $3d_{5/2}$ component of the NbN doublet in the pre-H*-exposed sample is at 204.3 eV binding energy. After 2 h H*-exposure, the doublet is shifted by ≈ 0.3 eV lower binding energy, attributed to de-nitridation. Note that following 2 h H* exposure, no significant shift in the fitted doublet is observed.

The N 1s spectra of the NbN sample were fitted with one peaks, with a GL set to 30. In the pre-exposed sample, the NbN/NbO_xN_y peak position is fitted at 397.1 eV binding energy.[9] Upon 2 h H*-exposure the peak shifted by 0.4 eV to a higher binding energy, attributed to surface de-oxidation. No further shift in the peak position is observed after 2 h H*-exposure.

FWHM of the fitted peaks and their respective areas are provided in the subsequent table (Table S3 and S4).

Table S3. FWHM and area in eV of the fitted peaks in the Nb3d and N 1s spectra of the pre-exp, 2 h H*-exp, and 4 h H*-exp NbN sample taken at $\Theta = 34.25°$. .

Peak	Pre-exp		2 h H*-exp		4 h H*-exp	
	FWHM	Area CPS	FWHM	Area CPS	FWHM	Area CPS
Nb $3d_{5/2}$ NbN_x	1.0	1111	1.0	1525	1.1	1640
Nb $3d_{5/2}$ NbO_xN_y	1.9	813	2.0	586	1.9	519
Nb $3d_{5/2}$ Nb_2O_5	2.0	461	2.0	276	2.0	253
N 1s NbN_x	1.3	717	1.2	585	1.2	564

Table S4. FWHM and area in eV of the fitted peaks in the Nb 3d and N 1s spectra of the 6 h H*-exp, 8 h H*-exp,and 10 h H*-exp NbN sample taken at $\Theta = 34.25°$.

Peak	6 h H*-exp		8 h H*-exp		10 h H*-exp	
	FWHM	Area CPS	FWHM	Area CPS	FWHM	Area CPS
Nb $3d_{5/2}$ NbN_x	1.0	1650	1.1	1702	1.2	1700
Nb $3d_{5/2}$ NbO_xN_y	1.9	521	1.8	458	1.8	458
Nb $3d_{5/2}$ Nb_2O_5	2.0	210	2.0	246	2.0	246
N 1s NbN_x	1.2	550	1.1	516	1.1	516

5. Low kinetic energy (LKE) and valence band (VB) spectra of the samples

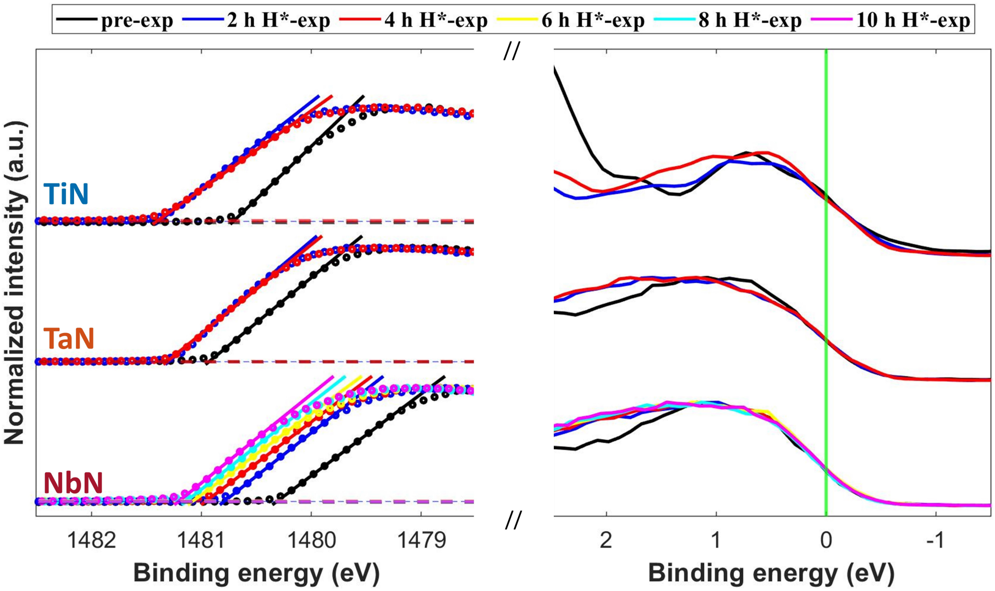

Figure S8. Low kinetic energy (LKE) and valence band (VB) spectra of the pre- and post-H*-exposed TiN, TaN, and NbN samples taken at a bias voltage of -16.4 V. To compensate for the bias voltage, VB spectra are also collected without any applied bias. The binding energies of the biased samples are adjusted so that the valence band spectra taken without bias and with bias overlap. Since in our instrument, the sample normal and the entrance of the electron analyzer are not parallel, there is a systematic deviation of -1.0±0.2 eV in the measured secondary electron cut-off. Furthermore, there is an uncertainty of ±0.1 eV in the measured secondary electron cutoff.

The work function is calculated using the following relation:

Work function (eV) = Al−Kα photon energy (1486.6 eV) - Fermi level (0 eV) - Secondary electron cutoff + systematic offset (-1 eV) ± uncertainty (0.3 eV)

6. Systematic deviation in the measured secondary electron cut-off

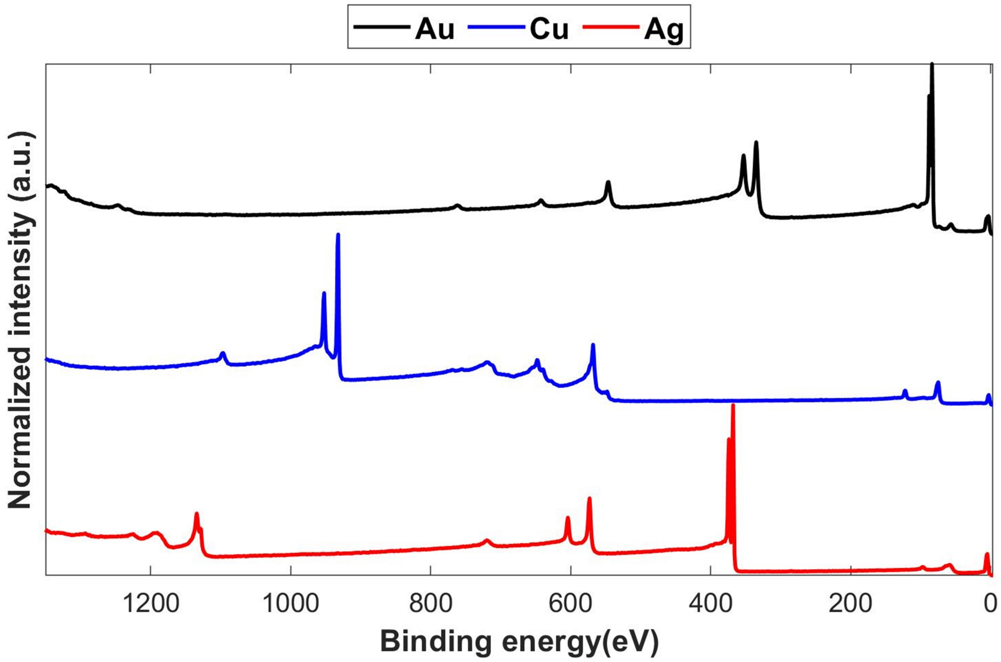

Figure S9. XPS survey spectra of Au, Cu, and Ag foils after 3 minutes of sputter cleaning with 1 keV Ar^+. No impurity was detected.

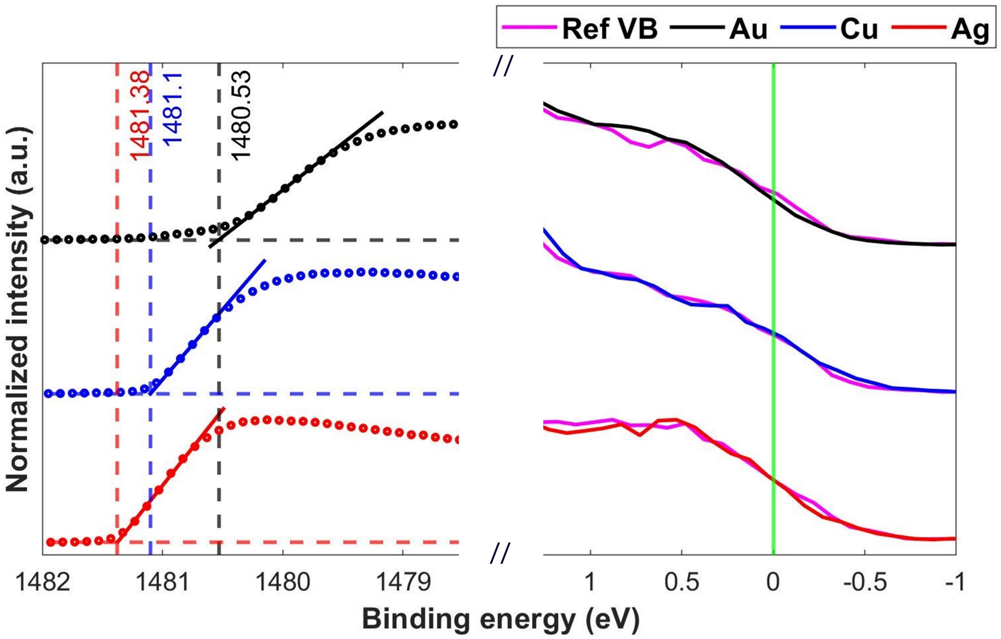

Figure S10. Low kinetic energy (LKE) and valence band (VB) spectra of Au, Cu, and Ag, taken at a bias voltage of -16.4 V. To compensate for the bias voltage, VB spectra are also collected without bias (Ref VB).

Table S5. Systematic offset between the measured and the reported work functions of Au, Cu and Ag is -1±0.2 eV.

Element	Secondary electron cut-off (eV)	Measured work function (eV)	Reported work function (eV)[?]	Offset (eV)
Au	1480.53±0.1	6.07±0.1	5.1±0.1	-0.97±0.2
Cu	1481.04±0.1	5.56±0.1	4.65±0.05	-0.91±0.15
Ag	1481.38±0.1	5.22±0.1	4.26	-0.96±0.1
			Offset	-1±0.2

References

(1) Chase, M. W. NIST-JANAF Thermochemical Tables 4th ed. *J. of Physical and Chemical Reffernce Data* **1998**, 1529–1564.

(2) Barin, I.; Platzki, G. *Thermochemical data of pure substances*; Wiley Online Library, 1989; Vol. 304.

(3) Greczynski, G.; Hultman, L. X-ray photoelectron spectroscopy: towards reliable binding energy referencing. *Progress in Materials Science* **2020**, *107*, 100591.

(4) Jaeger, D.; Patscheider, J. Single crystalline oxygen-free titanium nitride by XPS. *Surface Science Spectra* **2013**, *20*, 1–8.

(5) Greczynski, G.; Hultman, L. Self-consistent modelling of X-ray photoelectron spectra from air-exposed polycrystalline TiN thin films. *Applied Surface Science* **2016**, *387*, 294–300.

(6) Lamour, P.; Fioux, P.; Ponche, A.; Nardin, M.; Vallat, M.-F.; Dugay, P.; Brun, J.-P.; Moreaud, N.; Pinvidic, J.-M. Direct measurement of the nitrogen content by XPS in self-passivated TaNx thin films. *Surface and Interface Analysis: An International Journal*

devoted to the development and application of techniques for the analysis of surfaces, interfaces and thin films **2008**, *40*, 1430–1437.

(7) Kasatikov, S.; Filatova, E.; Sakhonenkov, S.; Konashuk, A.; Makarova, A. Relationship between Ta oxidation state and its local atomic coordination symmetry in a wide range of oxygen nonstoichiometry extent of TaO x. *The Journal of Physical Chemistry C* **2019**, *123*, 6849–6860.

(8) McLellan, R. A.; Dutta, A.; Zhou, C.; Jia, Y.; Weiland, C.; Gui, X.; Place, A. P.; Crowley, K. D.; Le, X. H.; Madhavan, T. et al. Chemical profiles of the oxides on tantalum in state of the art superconducting circuits. *Advanced Science* **2023**, *10*, 2300921.

(9) Havey, K.; Zabinski, J.; Walck, S. The chemistry, structure, and resulting wear properties of magnetron-sputtered NbN thin films. *Thin Solid Films* **1997**, *303*, 238–245.
